# Effective period of conservative treatment in patients with acute calcific periarthritis of the hand

**DOI:** 10.1186/s13018-018-0997-5

**Published:** 2018-11-14

**Authors:** Jihyeung Kim, Kee Jeong Bae, Do Weon Lee, Yo-Han Lee, Hyun Sik Gong, Goo Hyun Baek

**Affiliations:** 1 Department of Orthopaedic Surgery, Seoul National University Hospital, Seoul, South Korea; 2 Department of Orthopaedic Surgery, Seoul National University Boramae Medical Center, Seoul, South Korea; 3 Department of Orthopaedic Surgery, Seoul National University Bundang Hospital, Seongnam, South Korea

**Keywords:** Calcific periarthritis, Hand, Conservative treatment, NSAIDs

## Abstract

**Background:**

Acute calcific periarthritis of the hand is a relatively uncommon painful condition involving juxta-articular deposits of amorphous calcium hydroxyapatite. Although conservative treatments have been generally considered effective, there is little evidence regarding how long they could remain effective.

**Methods:**

We retrospectively reviewed ten patients who were diagnosed with acute calcific periarthritis of the hand from January 2015 to June 2018. We recommended the use of warm baths, nonsteroidal anti-inflammatory drugs (NSAIDs), and limited activity as initial treatments. If the pain persisted despite at least 3 months of conservative treatment, we explained surgical treatment options. If the pain improved, we recommended gradual range-of-motion exercises with the continuation of daily NSAIDs use. The visual analogue scale (VAS) score for pain at each subsequent visit (3, 6, and 9 months) was compared with that of the previous visit to investigate whether the pain had decreased during each time interval. Simple radiographs taken at each visit were compared with those taken at the previous visit to determine whether any significant changes in the amount of calcification had occurred during each time interval.

**Results:**

All 10 patients with 17 affected joints continued conservative treatments for an average of 11.1 months. The average VAS score for pain at the initial visit was 7, while that at 3, 6, and 9 months was 4.3, 3.3, and 2.9, respectively. There was a significant reduction in the VAS score at 3 and 6 months, but not at 9 months (*P* values = 0.004, 0.008, and 0.598, respectively). The simple radiographs also showed a significant reduction in the amount of calcification at 3 and 6 months, but not at 9 months (*P* values = 0.020, 0.034, and 0.083, respectively).

**Conclusions:**

Patients with acute calcific periarthritis of the hand exhibited residual pain and calcification for a relatively prolonged period. Those who continued conservative treatment, including NSAIDs, showed pain relief and reduced calcification for up to 6 months. These results suggest that conservative treatment could be tried for at least 6 months before considering the surgical treatment of calcific periarthritis of the hand.

## Introduction

Acute calcific periarthritis is an unusual and painful condition associated with juxta-articular deposits of amorphous calcium hydroxyapatite [1]. Calcific periarthritis has many other names, such as calcific tendinitis, calcium apatite deposition disease, hydroxyapatite deposition disease, calcific bursitis, and hydroxyapatite rheumatism [2]. It can occur primarily as an idiopathic entity or as a secondary process in end-stage renal disease, collagen vascular disease, vitamin D intoxication, or tumoral calcinosis [3]. In the upper extremities, acute calcium apatite deposition disease is exceedingly common in the shoulder, being present in approximately 3–8% of asymptomatic shoulders and 33–42% of shoulders with symptoms of subacromial pain syndrome [2]. Involvement of the hand and wrist is relatively uncommon, occurring in less than 2% of patients [2, 4]. It has been reported that the most common location within the hand and wrist is the flexor carpi ulnaris (FCU) tendon insertion site, occurring in 30–70% of the cases. [5, 6] Calcific periarthritis involving the metacarpophalangeal (MCP) joint or interphalangeal (IP) joint occurs less commonly, although one recent case series study reported more cases involving the MCP or IP joint than those involving the FCU insertion site [7].

Although common clinical manifestations of acute calcific periarthritis include the rapid onset of erythema, swelling, and restricted motion, patients generally suffer from the acute exacerbation of severe pain. As in other parts of the body, patients with acute calcific periarthritis of the hand typically complain of severe pain limited to the involved area of the hand. According to one previous report, over 25% of patients with acute calcific periarthritis of the hand visited the emergency department for intense pain [5]. A more recent study showed that 70% of patients with acute calcific periarthritis of the hand presented to the emergency department for the acute onset of severe night pain [6].

Conservative management strategies, including rest, splinting, and pain relievers, have been known to be effective for the treatment of acute calcific periarthritis [1]. Nonsteroidal anti-inflammatory drugs (NSAIDs) have been reported to alleviate symptoms and even to assist in reducing the amount of calcification [1]. Resolving pain and reducing calcification using conservative treatment can take from several days to several weeks [1, 2, 6, 8]. In uncommon cases of residual or persistent pain after initial conservative treatment, other treatment modalities, such as local injections, extracorporeal shockwave therapy (ESWT), platelet-rich plasma (PRP) therapy, and needling, have been applied [2, 8]. Several authors have reported rapid pain relief achieved by local anesthetic injections with or without steroids in acute calcific periarthritis of the hand [6, 9]. However, there is insufficient evidence regarding how long conservative treatment should be continued before considering other treatment modalities [2, 8]. Thus, we conducted this retrospective study of patients with acute calcific periarthritis of the hand to investigate how long conservative treatment could be effective.

## Methods

We retrospectively reviewed patients who had visited the clinic and had been diagnosed with acute calcific periarthritis of the hand from January 2015 to June 2018. The diagnosis was made based on both the acute onset of periarticular pain within 1 month and periarticular calcific deposits visible on simple radiographs. Patients with calcific periarthritis of the MCP joint and IP joint were included, while those with calcific periarthritis of the radiocarpal, distal radioulnar, intercarpal, and carpometacarpal joints were excluded. Among the patients with acute calcific periarthritis of the hand, those with more than 6 months of follow-up were included. Ten patients were eligible for inclusion criteria. The mean patient age was 49.4 ± 9.2 years (range, 32–64 years); one (10%) patient was male and nine (90%) patients were females. Five patients showed the involvement of multiple joints, so a total of 17 joints were involved in 10 patients. Three (30%) patients recalled a previous minor trauma to the involved joints within the past 6 months. Five (50%) patients reported the current use of medications for other diseases: three (30%) patients were being treated with anti-hypertensive drugs, two (20%) with cholesterol-lowering agents, and two (20%) with oral hypoglycemic agents. There were no patients with underlying disease, such as end-stage renal disease or collagen vascular disease. Initial laboratory test results, including those of calcium, phosphorus, and alkaline phosphatase (ALP), showed no abnormalities, except in one patient, who had a high ALP level of approximately 205 IU/L (Table 1). Among the 17 joints, 9 (52.9%) were proximal interphalangeal (PIP) joints, 7 (41.2%) were MCP joints, and 1 (5.9%) was the IP joint of the thumb. There was no distal interphalangeal (DIP) joint involvement. All five fingers were affected with no definite tendency (Table 2).

**Table 1:** Demographic data of the patients

Patient number	Sex	Age	Location	Duration of conservative treatment (months)	Previous trauma history	Medical comorbidity	Initial laboratory abnormality
1	F	54	Rt. 2nd MCP, Lt. 1st MCP	6	–	Hypertension	–
2	F	64	Rt. 1st IP Lt. 5th PIP	9	–	Dyslipidemia	–
3	F	52	Rt. 2nd MCP	6	+	Hypertension Dyslipidemia	–
4	F	39	Rt. 3rd PIP B/L 4th PIP	9	–	–	Elevated ALP
5	F	58	Lt. 1st MCP B/L 5th MCP	9	–	–	–
6	F	46	Rt. 3rd PIP	9	+	–	–
7	M	32	Lt. 1st MCP, Lt. 2nd PIP	12	–	–	–
8	F	52	Rt. 3rd PIP	9	–	Hypertension DM	–
9	F	46	Rt. 2nd PIP	18	+	DM	–
10	F	51	Lt. 2nd PIP	24	–	–	–
Mean		49.4 ± 9.2		11.1 ± 5.4			

*Rt* right, *Lt* left, *B*/*L* bilateral, *MCP* metacarpophalangeal, *PIP* proximal interphalangeal, *IP* interphalangeal, *DM* diabetes mellitus, *ALP* alkaline phosphatase

**Table 2:** Locations of 17 joints involved according to joint level and finger

	Location	Number	Percentage (%)
Joint level	MCP joint	7	41.2
	PIP joint	9	52.9
	DIP joint	0	0
	Thumb IP joint	1	5.9
Finger	Thumb	4	23.5
	Index finger	5	29.4
	Long finger	3	17.6
	Ring finger	2	11.8
	Little finger	3	17.6

*MCP* metacarpophalangeal, *PIP* proximal interphalangeal, *DIP* distal interphalangeal, *IP* interphalangeal

For the initial 3 months, we combined the use of hot packs or warm baths with NSAIDs and limited exercises. We recommended continuing conservative treatment for at least 3 months. If pain persisted without improvement or aggravated after at least 3 months of conservative treatment, we explained surgical treatment options and allowed the patients to choose whether to undergo surgical treatment or to continue conservative treatment. If there was some improvement in the symptoms, we recommended gradual range-of-motion exercises of the affected joints while continuing the use of NSAIDs.

At each follow-up visit, the pain score was recorded routinely. The pain score was measured using a visual analog scale (VAS) system. The VAS score at the 3, 6, and 9 month follow-up visits were compared with the VAS score at the previous (initial, 3 months, and 6 months) visit using a paired *t* test to investigate whether the pain had decreased during each time interval.

Simple radiographs of each affected joint were acquired at each follow-up visit. The amount of calcification at each visit was compared with that at the previous visit to determine whether any significant change had occurred. The radiographic change in the amount of calcification was categorized as follows: maintenance of complete resolution, decrease with complete resolution, decrease with residual calcification, no change with persistent calcification, and increase. All radiographs were assessed by two of the authors, who are orthopedic surgeons and were blinded to all other subject information. Intra-observer reliability was evaluated using a repeated assessment after 4 weeks. Inter-observer reliability was evaluated using the assessments of the two examiners. The intra-observer and inter-observer reliabilities were evaluated using kappa coefficients. We determined whether there had been a significant reduction in calcification during each time interval using the Wilcoxon signed-rank test. All statistical analyses were performed using SPSS ver. 20.0 (SPSS Inc., Chicago, IL, USA).

## Results

**Fig. 1:**
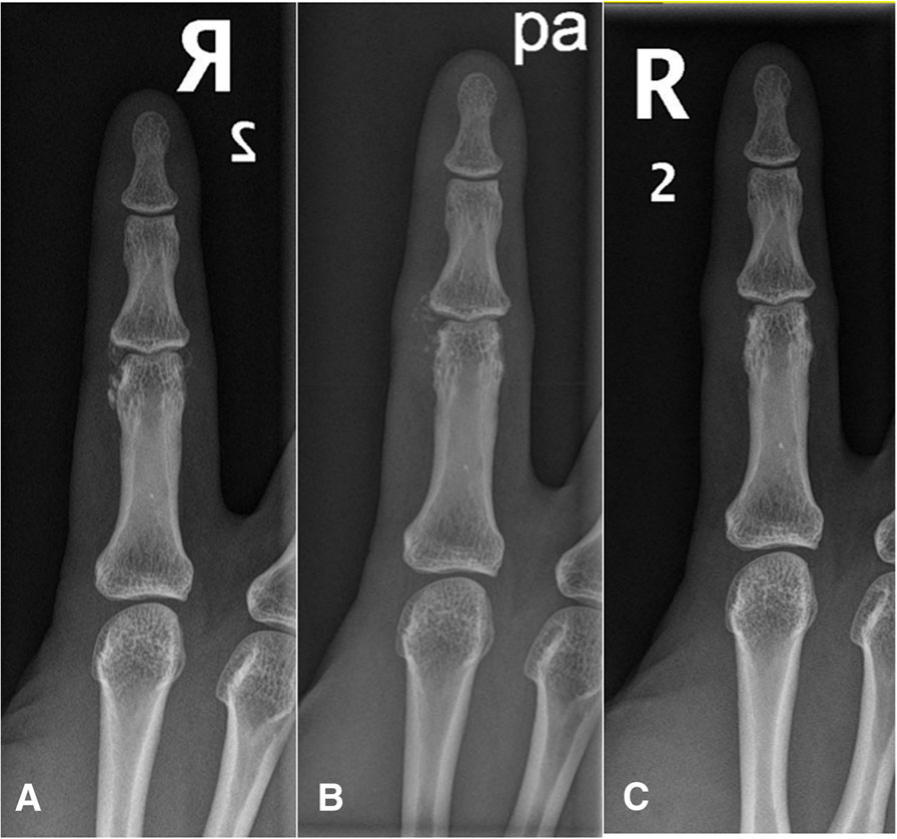
Patient (#9) with calcific periarthritis involving the PIP joints of the right second finger continued conservative treatment for 18 months and was pain-free at the last follow-up. X-rays taken at the initial visit (**a**) and at the 9- (**b**) and 18-month (**c**) follow-ups showed a marked reduction in calcification

All ten patients continued the conservative treatments until the last visit (Fig. 1). The average duration of conservative treatment was 11.1 ± 5.4 months (range, 6–24 months). Among the ten patients, eight patients showed marked improvement of in the VAS score for pain after the initial 3 months of conservative treatment, while two patients (patients #1 and #5) showed persistently high VAS scores. We explained the surgical treatment options for these two patients. However, they chose to continue conservative treatments because the frequency and duration of painful events had decreased after conservative treatment. They did not change their minds until the last visit.

**Fig. 2:**
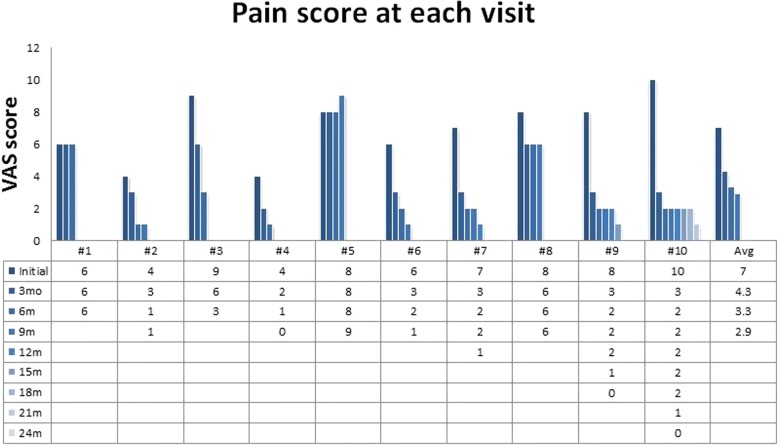
VAS scores for pain during the entire follow up period

The average VAS score at the initial visit was 7, while that at 3, 6, and 9 months was 4.3, 3.3, and 2.9, respectively. There was a significant reduction in the VAS score from the initial visit to the 3-month follow-up and from the 3-month follow-up to the 6-month follow-up (*P* values = 0.004 and 0.008, respectively). However, there was no significant change in the VAS score between the 6- and 9-month follow-ups (*P* value = 0.598) (Fig. 2).

Regarding the radiographic assessment, the kappa coefficient for the intra-observer reliability was 0.854 for one examiner and 0.832 for the other examiner. The kappa coefficient for the inter-observer reliability was 0.818. Therefore, the radiographic assessments of only one examiner were used in the analysis, given the high inter-observer reliability. Simple radiographs at the 3-month follow-up showed a decrease in the amount of calcification in 8 (47%) out of the 17 joints, including 4 (24%) joints with complete resolution, 8 (47%) joints with no change, and 1 (6%) joint with increased calcification. The radiographic assessments performed at 6 months showed resolution maintenance in four (24%) joints and decreased calcification in seven (41%), including two (12%) with complete resolution, while no change was observed in five (29%) joints and increased calcification was observed in one (6%) joint. At 9 months, three (18%) joints were lost to follow-up, five (29%) joints showed maintenance resolution, and three (18%) joints showed decreased calcification, including two (12%) with complete resolution, while no change occurred in six (35%) joints. There was a significant reduction in the amount of calcification at the 3- and 6-month follow-ups (*P* values = 0.020 and 0.034, respectively), but not at the 9-month follow-up (*P* value = 0.083) (Table 3).

**Table 3:** Radiographic change in the amount of calcification

Patient number	Joint number	Joint location	3Mo	6Mo	9Mo	12Mo	15Mo	18Mo	21Mo	24Mo
1	1	Rt. 2nd MCP	D	D						
	2	Lt. 1st MCP	D	N						
2	3	Rt. 1st IP	N	N	N					
	4	Lt. 5th PIP	N	N	N					
3	5	Rt. 2nd MCP	R	M						
4	6	Rt. 3rd PIP	R	M	M					
	7	Rt. 4th PIP	N	D	N					
	8	Lt. 4th PIP	N	N	N					
5	9	Rt. 5th MCP	N	N	N					
	10	Lt. 1st MCP	N	D	N					
	11	Lt. 5th MCP	I	I	D					
6	12	Rt. 3rd PIP	D	R	M					
7	13	Lt. 1st MCP	R	M	M	M				
	14	Lt. 2nd PIP	R	M	M	M				
8	15	Rt. 3rd PIP	N	D	D					
9	16	Rt. 2nd PIP	N	D	D	D	D	R		
10	17	Lt. 2nd PIP	N	R	M	M	M	M	M	M

*Mo* months, *Rt* right, *Lt* left, *MCP* metacarpophalangeal, *IP* interphalangeal, *PIP* proximal interphalangeal, *M* maintenance of complete resolution, *R* decrease with complete resolution, *D* decrease with residual calcification, *N* no change with persistent calcification, *I* increase in calcification

## Discussion

In this study, we investigated the effective period of conservative treatment, including NSAIDs, for acute calcific periarthritis of the hand. According to previous reports, the symptomatic relief of acute calcific periarthritis can take from several days to several weeks, and reducing calcification takes several weeks [1, 2, 6, 10]. However, our data show that patients with acute calcific periarthritis of the hand suffered from pain for a relatively prolonged period: the average VAS score was 4.3 at 3 months and 3.3 at 6 months. Radiographic assessments also revealed residual calcification in 13 (76%) out of 16 affected joints at 3 months and in 11 (64%) joints at 6 months. Nevertheless, the results of this study suggest that those who continued conservative treatment experienced significant pain relief and calcification reduction at the 3- and 6-month follow-ups, but not at the 9-month follow-up.

Acute calcific periarthritis of the hand is known to mainly affect premenopausal women, with a female-to-male ratio of 5:1 [6, 11]. This female predilection of the disease was observed in this study, as nine of the ten patients were women. Regarding the involved joints, MCP joints had been known as the most commonly involved, followed by PIP joints [7, 10]. In our study, the PIP and MCP joints were commonly affected, while there was no DIP joint involvement.

Hamada et al. have previously suggested that the clinical course depends on the location of calcific deposits in the shoulder [12]. They found that calcium deposits in the tendon resulted in more chronic symptom than deposits in the bursa. One previous study by Kim and Park comparing acute calcific periarthritis and peritendinitis in the hand and wrist also revealed distinctive clinical features according to the location of calcific deposits [7]. They found that patients with acute calcific periarthritis showed involvement entirely in the hand area, such as the MCP and PIP joints, whereas those with acute calcific peritendinitis showed involvement entirely in the wrist area, such as the FCU insertion. They also showed that the recurrence rate was higher in the peritendinitis group, whereas there was no recurrence after conservative treatment in the periarthritis group. The results of our study indicate that all calcific deposits involving the hand presented as periarthritis, not as peritendinitis, and no recurrence occurred during the mean follow-up period of 11 months. Although further studies with a prospective design, control group, and large sample size are required to elucidate the nature of acute calcific periarthritis, we speculate that calcific deposits in the hand mostly presenting as periarthritis might show clinical features distinct from those in other sites mostly presenting as peritendinitis.

Two patients (patients #1 and #5) showed no reduction in the VAS score even after 6 months of conservative treatment. We could not identify any distinct clinical features from the medical records or simple radiographs of these two patients. The patients did not have any previous history of trauma, laboratory abnormalities, or current medications, except one patient was under treatment with anti-hypertensive drugs. Some authors have recommended other treatment modalities, such as local injections, ESWT, PRP therapy, and needling, for patients with residual or persistent pain after conservative treatment [2, 8]. However, there is insufficient qualifying evidence to support the superiority of these treatment modalities over conservative treatment [2, 8]. The results of our study show that there was pain reduction for up to 6 months in patients who continued conservative treatment.

Surgical treatments are usually indicated for calcific periarthritis with recurrent or persistent pain even after nonsurgical treatments [4, 8]. However, in our study, no patients opted for surgical treatment after the initiation of conservative treatment, although we explained the surgical options to two patients with persistently high VAS scores. One previous study showed that one (8%) patient with calcific peritendinitis of the wrist needed surgery for recurrent symptoms, while no patients among 17 patients with calcific periarthritis of the hand needed surgery [7]. The results of our study suggest that conservative treatment could be tried for at least 6 months before considering surgical treatments.

The exact pathogenesis of calcific periarthritis remains controversial. In a relatively recent proposal by Uhthoff and colleagues, hypoxia in critical areas of the tendon initiates calcific periarthritis, followed by fibrocartilaginous metaplasia of the tendon [13]. They divided the natural progression of the disease into four phases: the precalcific, formative, resorptive, and postcalcific phases [14]. In the precalcific phase, collagen fibers of the tendon undergo metaplasia to form fibrocartilage tissue. During the formative phase, calcified apatite crystals are developed. In the resorptive phase, leukocytes, lymphocytes, and giant cells gather to form a calcium granuloma. Then, new capillaries and collagen fibers form during the postcalcific phase [14]. It is believed that the rupture of these pre-existing calcific deposits followed by an inflammatory response leads to the acute onset of clinical symptoms [6]. Although the specific role of NSAIDs in reducing pain or calcification in calcific periarthritis has not yet been clarified, it is assumed that NSAIDs mitigate the inflammatory process [8].

Several limitations of this study should be noted. First, above all, there was no control group. Thus, we cannot be sure whether the conservative treatments actually altered the natural course of calcific periarthritis. Second, this was a retrospective study with limited clinical data. There were no additional clinical data available during the 3-month intervals. With more weekly or at least monthly data, more details regarding reductions in pain and calcification might be elucidated. Third, the small sample size limits the generalizability of the results of this study. A comparative analysis, such as between a high- and low-response group and between a surgical and conservative treatment group in a relatively large cohort, may reveal significantly distinct clinical features, although such comparisons could not be performed in this study due to the small sample size.

## Conclusions

Patients with acute calcific periarthritis of the hand revealed residual pain and calcifications for a relatively prolonged period and those who continued conservative treatment, including NSAIDs, showed pain relief and reduced calcification up to the 6-month follow-up. These results suggest that conservative treatment could be tried for at least 6 months before considering surgical options for treatment of calcific periarthritis of the hand. Further studies with a prospective design, large sample size, and control group are required to clarify the optimal treatments for calcific periarthritis.

## Abbreviations

DIP: Distal interphalangeal
IP: Interphalangeal
MCP: Metacarpophalangeal
NSAIDs: Nonsteroidal anti-inflammatory drugs
PIP: Proximal interphalangeal
VAS: Visual analogue scale
